# Bioactive lipid signaling and lipidomics in macrophage polarization: Impact on inflammation and immune regulation

**DOI:** 10.3389/fimmu.2025.1550500

**Published:** 2025-02-14

**Authors:** Juan P. Rodríguez, Javier Casas, María A. Balboa, Jesús Balsinde

**Affiliations:** ^1^ Laboratorio de Investigaciones Bioquímicas de la Facultad de Medicina (LIBIM), Instituto de Química Básica y Aplicada del Nordeste Argentino (IQUIBA-NEA), Universidad Nacional del Nordeste, Consejo Nacional de Investigaciones Científicas y Técnicas (UNNE-CONICET), Corrientes, Argentina; ^2^ Instituto de Biología y Genética Molecular, Consejo Superior de Investigaciones Científicas Uva, Valladolid, Spain; ^3^ Departamento de Bioquímica y Biología Molecular y Fisiología, Universidad de Valladolid, Valladolid, Spain; ^4^ Centro de Investigación Biomédica en Red de Diabetes y Enfermedades Metabólicas Asociadas (CIBERDEM), Instituto de Salud Carlos III, Madrid, Spain

**Keywords:** macrophage phenotype, lipidomic profiling, phospholipase a2 signaling, lipid remodeling, inflammaging

## Abstract

Macrophages, crucial innate immune cells, defend against pathogens and resolve inflammation, maintaining tissue balance. They perform phagocytosis, present antigens to T cells, and bond innate and adaptive immunity through various activation states. Classical activation is associated with Th1 responses and interferon γ production, while alternative activation, induced by interleukin 4, is characterized by increased endocytosis, reduced secretion of pro-inflammatory cytokines, and roles in immunoregulation and tissue remodeling. Although these represent opposite extremes observed *in vitro*, the remarkable plasticity of macrophages allows for a wide spectrum of activation phenotypes that are complex to characterize experimentally. While the application of omics techniques has resulted in significant advances in the characterization of macrophage polarization, lipidomic studies have received lesser attention. Beyond their role as structural components and energy sources, lipids function as signaling molecules that regulate macrophage activation and polarization, thereby shaping immune responses. This work reviews the interaction between lipid signaling and macrophage polarization, exploring how lipid metabolism influences macrophage phenotype and function. These insights offer potential therapeutic strategies for immune-mediated diseases and inflammation-related disorders, including inflammaging.

## 1 Introduction

Macrophages are tissue-resident cells that act as sentinels of the immune system, regulating a delicate balance between defense against pathogens and initiation/resolution of inflammation, thus maintaining tissue homeostasis (1–3). Macrophages exhibit a high degree of plasticity and diversity. It is widely acknowledged that these cells originate from progenitors starting from embryogenesis onwards and continuing throughout an individual’s lifespan. Many tissue-resident macrophages are derived from embryonic progenitors and are capable of self-renewal without relying on adult blood monocytes. In steady-state conditions, fetal-derived and monocyte-derived macrophages co-exist, both contributing to tissue homeostasis. During inflammation, monocytes are recruited to generate macrophages that play crucial roles in either promoting local inflammation or facilitating its resolution (4–6). This guarantees a permanent presence of macrophages in adult tissues through self-renewal, and increases in their number during inflammatory circumstances (7, 8).

The concept of classical macrophage activation was first introduced by Mackaness (9) to describe the antigen-dependent but nonspecific enhanced microbicidal activity of macrophages following secondary pathogen exposure. This activity, later linked to Th1 (CD4+ T helper lymphocytes) responses and interferon γ (IFNγ) production, was also associated with cytotoxic and antitumoral properties (9, 10). In contrast, IFNγ was found to inhibit the expression of the macrophage mannose receptor, while interleukin 4 (IL4) enhanced its expression and induced an alternative activation, characterized by increased endocytic clearance and reduced pro-inflammatory cytokine secretion (11, 12). Mills and colleagues (13–15) later proposed the M1/M2 classification, observing that macrophages from *Leishmania major*–resistant Th1 mouse strains produced more nitric oxide when activated by IFNγ or bacterial lipopolysaccharide (LPS) compared to macrophages from Th2 strains, which metabolized arginine to ornithine instead. Since nitric oxide inhibits cell division and ornithine promotes it, the M1 and M2 phenotypes were hypothesized to have opposing roles in inflammation. Over time, research has revealed a spectrum of macrophage activation states between M1 and M2, depending largely on the type of stimuli (16, 17). Today, M1 macrophages are recognized as classically activated (LPS plus IFNγ), pro-inflammatory cells that express inducible nitric oxide synthase (iNOS) and produce pro-inflammatory cytokines, initiating inflammation. In contrast, M2 macrophages are alternatively activated (IL4 or IL13), anti-inflammatory/reparative cells that express high levels of arginase and produce anti-inflammatory cytokines, promoting the resolution of inflammation.

Macrophage polarization leads to notable alterations in gene expression, precluding the definition of a specific activation state by a single gene. This uncertainty is often mitigated by using multiple markers to characterize activation outcomes. Many laboratories have expanded marker assignments to include transcription factors, cytokines, chemokines, and cell surface markers, aiming for a comprehensive understanding of macrophage activation (18–21). These aspects highlight the complexity of macrophage polarization and the need to refine our understanding of this paradigm beyond conventional approaches. The shift from traditional gene and protein markers toward omics approaches, such as transcriptomics and proteomics, has improved our understanding of macrophage activation (22–25). However, lipidomics has received lesser attention. Beyond their traditional roles as structural components and energy sources, lipids function as signaling molecules that influence numerous cellular processes (26, 27). In fact, lipid metabolism and signaling pathways have emerged as key regulators of macrophage activation and polarization, thereby shaping immune responses and inflammatory outcomes (28). In this review, we comprehensively explore the sophisticated interplay between lipid signaling and macrophage polarization, focusing on how lipid metabolism influences macrophage phenotype and function and vice versa.

## 2 Intermediary metabolism and its influence on macrophage polarization

### 2.1 Immunometabolism

**Figure 1:**
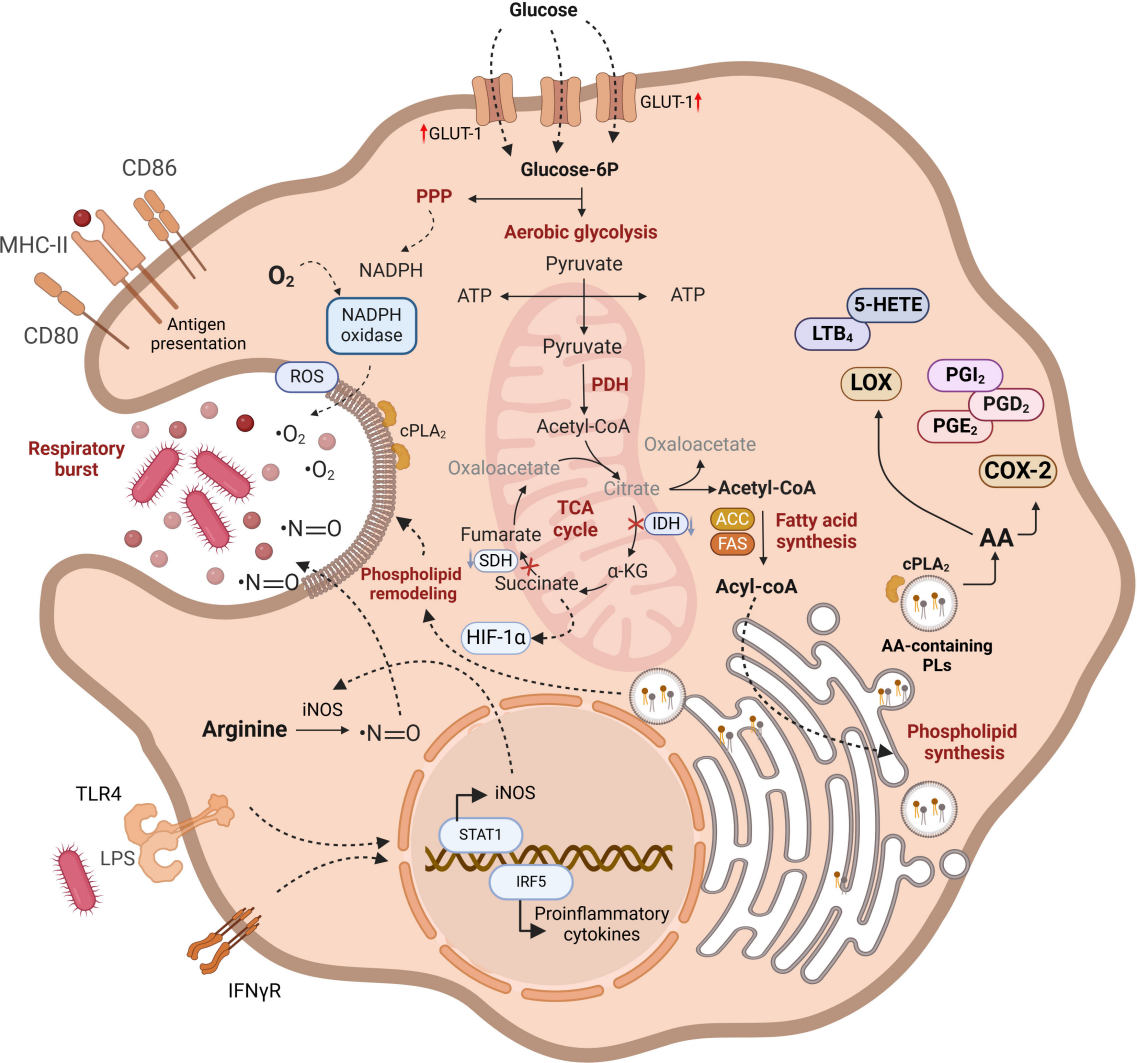
Functional characteristics of M1 macrophages. Lipid metabolism plays a crucial role in the metabolic and functional characteristics of M1 macrophages. Increased glucose uptake via GLUT-1 enhances glycolysis, but glucose-6P is also diverted to the pentose phosphate pathway (PPP) for NADPH and ROS production. Disruptions in the TCA cycle lead to the mitochondrial export of citrate and succinate, with citrate being converted into acetyl-CoA, which is pivotal for the synthesis of acyl-CoA and phospholipids. These lipids are vital for membrane remodeling during phagosome formation. TLR4 and IFNγR signaling enhance proinflammatory cytokine production and iNOS synthesis, contributing to ROS generation. Free arachidonic acid arising from phospholipid hydrolysis serves as a substrate for cyclooxygenase-2 and 5-lipoxygenase (5-LOX), resulting in the production of proinflammatory eicosanoids. Lipid pathways are in bold highlighting the importance of lipid metabolism in maintaining M1 macrophage functionality. AA-containing phospholipids are produced from preexistig phospholipids via deacylation/reacylation reactions. For further details see text.

**Figure 2:**
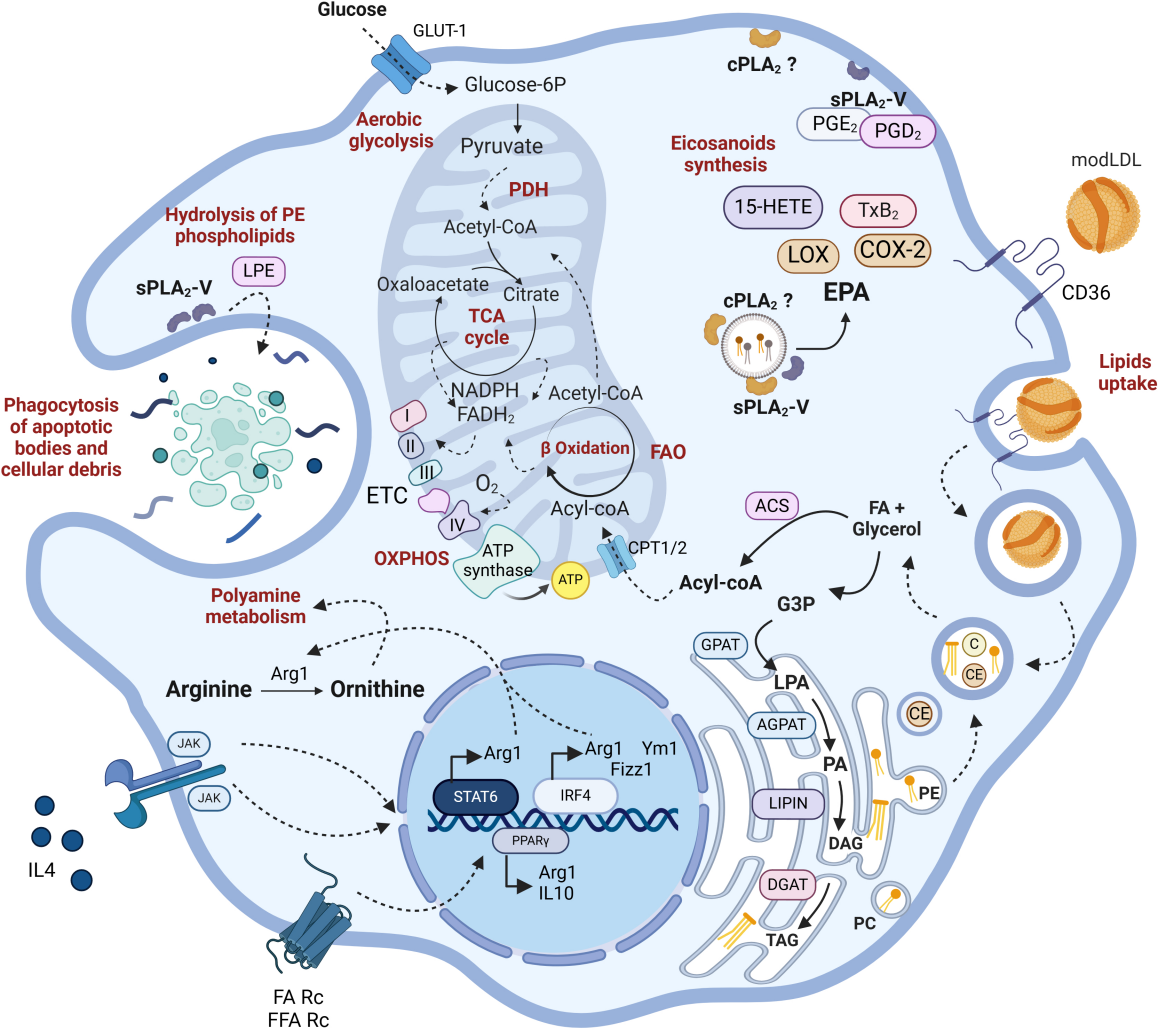
Lipid metabolism in M2 macrophages. IL4R signaling leads to the upregulation of typical M2 markers such as Arg1, YM1, or Fizz1. Additionally, PPARγ is upregulated by receptor-mediated signaling stimulated by free FA (FFA) or IL4 itself. M2 macrophages possess fully functional glycolytic and TCA cycles in contrast to M1. They metabolize arginine to produce ornithine and other polyamines that promote cell proliferation. They exhibit a high rate of phagocytosis focused on degrading cellular debris, dead bacteria, and opsonized particles. Through CD36-mediated endocytosis, they internalize modified LDLs, whose lipids serve for membrane remodeling, fueling FAO, and ETC. While the involvement of sPLA_2_-V in phagocytosis, via generation of ethanolamine lysophospholipids (LPE) has been well established, the role of cPLA_2_α is less characterized. Arg1, arginase-1; Chi3l3, chitinase-like protein 3; Fizz1, found in inflammatory zone-1 (also known as resistin-like alpha, Retnla); PPARγ, peroxisome proliferator-activated receptor γ. For further details see text.

Energy production is crucial for the proper functioning of cells, whether they are in a resting state or activated. Cells primarily generate energy through two key pathways: glycolysis and oxidative phosphorylation (OXPHOS). Similar to other normal cells, macrophages in their resting state largely rely on mitochondrial OXPHOS and catabolic metabolism to meet their energy requirements. However, when activated and, especially during classical activation, there is an increase in aerobic glycolysis, and reduction of OXPHOS, even in the presence of adequate oxygen levels. This phenomenon is referred to as metabolic reprogramming or Warburg effect (29). Studies on immunometabolism have demonstrated that metabolic reprogramming not only governs macrophage phenotype, but also influences their plasticity, depending on the primary immune specific function they need to fulfill (30, 31). It is clear that an interplay exists between the phenotype and the metabolic energy status of macrophages. Several high-quality review articles have been published on this topic, and the interested reader is kindly referred to them (28, 31–37). **Figures 1**, **2** provide a summary of these contributions, highlighting their connection to lipid metabolism and positioning acetyl-CoA and acyl-CoA at the core of intermediary metabolism. Notably, the origin and fate of acetyl-CoA are key factors that help distinguish between M1 and M2 (38).

In a nutshell, pro-inflammatory M1 macrophages are metabolically characterized by a high reliance on aerobic glycolysis and present two key breaks in the TCA cycle, leading to the accumulation of citrate, succinate, and itaconate. The excess succinate contributes to the stabilization of HIF1α, which subsequently activates the transcription of glycolytic genes, thereby sustaining the glycolytic metabolism in M1 macrophages. Additionally, M1 macrophages express high levels of iNOS, which produces nitric oxide from arginine. Nitric oxide, a free radical with microbicidal properties, inhibits the ETC, further reducing mitochondrial respiration. Additionally, M1 macrophages show increased production of ROS and fatty acid synthesis (**Figure 1**). In contrast, M2 macrophages are distinguished by their expression of the enzyme arginase, which catalyzes the hydrolysis of arginine into ornithine and urea, reducing the availability of arginine for nitric oxide production (**Figure 2**). M2 macrophages typically depend on OXPHOS, with an intact TCA cycle that provides substrates necessary for the ETC.

### 2.2 Fatty acid metabolism in polarized macrophages

Macrophages incorporate fatty acids (FA) from lipoprotein lipids via specific or scavenger receptors. In addition, they possess all the machinery necessary to synthesize FA via *de novo* pathways. Synthesis occurs in the cytosol using acetyl-CoA that is produced by ATP-citrate lyase (ACLY). This enzyme acts on the citrate that was originally generated in the mitochondria by the TCA cycle. Citrate leaves the organelle in exchange for malate in a process known as the citrate shuttle (**Figure 1**). Cytosolic acetyl-CoA is transformed to malonyl-CoA by acetyl-CoA carboxylase (ACC), participating in the synthesis of FA by repetitive additions of two carbons, catalyzed by fatty acid synthase (FAS), until the generation of a 16-carbon fatty acid, palmitic acid. Acetyl-CoA is also used for the synthesis of cholesterol in a complex multistep cytosolic pathway that is known as the mevalonate pathway. FA can be esterified to glycerol-3-phosphate or cholesterol to ultimately form phospholipids, triacylglycerols or cholesterol esters. Conversely, FA can be β-oxidized in the mitochondrial matrix, generating energy for the cell in a process that takes place through a repetitive cyclic series of reactions catalyzed by acyl CoA dehydrogenase, enoyl CoA hydratase, β-hydroxyl acyl CoA dehydrogenase, and β-keto thiolase. To enter the mitochondrial matrix, FA are transformed to fatty acyl-CoA by the fatty acyl-CoA synthetase and cross mitochondrial membranes using the transporters carnitine palmitoyl transferase I (CPT1) and carnitine palmitoyl transferase II (CPT-2) located in the outer membrane and the inner membrane respectively (**Figure 2**).

After palmitic acid is synthesized by FAS, FA diversity is created by the action of elongases and desaturases, which supply the cells with a varied number of FAs for membrane phospholipid synthesis and neutral lipid formation. FA desaturation and elongation enzymes are integral to macrophage physiology, influencing membrane composition, signaling pathways, and functional responses. Desaturases introduce double bonds into FA chains, altering their degree of unsaturation, while elongases extend the carbon chain length of FAs (39). These modifications are crucial for maintaining the fluidity and integrity of cellular membranes, which in turn affect receptor function and signal transduction in macrophages (40, 41). For instance, the activity of stearoyl-CoA desaturase 1, the enzyme that converts palmitic acid to palmitoleic acid, and stearic acid to oleic acid, has been implicated in modulating lipid composition, thereby influencing macrophage activation and inflammatory responses (42).

The balance between saturated and unsaturated FAs, regulated by both desaturases and elongases, appears to be essential for macrophage polarization. Alterations in desaturase activity can skew macrophage polarization, impacting immune responses and disease progression. In this regard, it has been shown that impairment of systemic docosahexaenoic acid (DHA, 22:6n–3) synthesis, which involves desaturase and elongase activities, affects macrophage plasticity and polarization (43). Thus, the absence of the elongase Elovl2 results in the increased expression of M1 markers in both M1 and M2 macrophages, and the reduced expression of M2 markers in M2 macrophages. Consequently, this elongase plays a crucial role in regulating the balance between pro-inflammatory and anti-inflammatory processes. Importantly, DHA supplementation in animals counteracts these effects (43). Other studies found that changes in FA desaturation were associated with insulin resistance, highlighting the broader implications of these enzymes in metabolic regulation (44). Moreover, the interplay between FA metabolism and macrophage function extends to disease contexts such as idiopathic pulmonary fibrosis. Dysregulation of FA elongation and desaturation pathways has been observed in this disorder, suggesting that targeting these metabolic processes could offer therapeutic avenues (45).

Numerous studies have described specific roles for FAs in regulating the inflammatory response of macrophages. Saturated FA such as palmitic acid and stearic acid, when in excess, generally promote inflammation and are associated with type 1 immune responses (46). In contrast, monounsaturated fatty acids such as oleic acid and palmitoleic acid can have anti-inflammatory effects, aligning with type 2 immune responses (47–49). Mammalian cells cannot synthesize long-chain polyunsaturated FAs (PUFA) of the n–6 series, (*e.g*. arachidonic acid, AA, 20:4n–6), or those of the n-3 series (*e.g*. DHA, 22:6n–3) *de novo*. However, they can produce them through the elongation and desaturation of their essential precursors, linoleic acid (18:2n–6) and α-linolenic acid (18:3n–3), which must be obtained from dietary sources. Because of the key roles of PUFAs in immune cell function, dietary habits significantly influence macrophage polarization. Several lines of research suggest that PUFAs of the n–3 series promote an M2 anti-inflammatory macrophage phenotype, which aids in tissue repair and resolution of inflammation (50–52). High dietary intake of n–3 PUFAs enhances M2 macrophage polarization and chemotaxis, thereby regulating tissue inflammation and metabolic health (53–56). Whether these effects are mediated by the FA itself or an oxygenated metabolite(s) is currently a matter of strong debate (57, 58). Conversely, diets high in n–6 PUFAs, which are abundant in animal fats, may predispose macrophages toward a pro-inflammatory M1 state (50–52). This shift can contribute to chronic inflammatory conditions. For example, a positive correlation has been found between the consumption of animal fats and the presence of pro-inflammatory macrophages in human adipose tissue, suggesting that dietary FA composition directly affects macrophage behavior (54–56, 59, 60). Therefore, maintaining a balanced intake of n–3 and n–6 PUFAs may be crucial for modulating macrophage polarization and supporting optimal immune function.

During proinflammatory activation of macrophages (TLR4 activation by LPS), AMPK activity decreases. As a consequence, phosphorylation of ACC is reduced, which increases its activity and promotes the conversion of acetyl-CoA to malonyl-CoA. Since malonyl-CoA is an inhibitor of CPT-1, M1 macrophages display reduced FAO and increased lipogenesis (61). In fact, lipid biogenesis, coupled with aerobic glycolysis, drives in M1 macrophages a metabolic shift akin to the Warburg effect (**Figure 1**). The source of acetyl-CoA necessary for FA synthesis in M1 macrophages is the glycolysis. As in the rest of the cells, glycolysis generates pyruvate from glucose that can be converted to lactate and secreted, or undergo decarboxylation in the mitochondria to render acetyl-CoA. Acetyl-CoA enters the TCA as citrate after condensation with oxaloacetate (OAA). Because in M1 macrophages there is a reduced expression of the TCA cycle enzyme isocitrate dehydrogenase, citrate increases in mitochondria and is shuttled to the cytosol to be used for lipid synthesis (62, 63). The mitochondrial citrate carrier, responsible for the transport of citrate from the mitochondria to the cytosol, and the following three cytosolic key enzymes participating in FA synthesis, namely ACLY, ACC and FAS, have all been implicated in LPS-induced inflammatory processes by participating in the production of NO, ROS and prostaglandins (CIC and ACLY); inducing epigenetic remodeling through promotion of histone acetylation (ACLY); helping during an early metabolic switch to glycolysis, and remodeling of the macrophage lipidome (ACC); altering the cell membrane order and composition, and disrupting Rho GTPase signaling, which is crucial for cell adhesion, migration, and activation (FAS) (63–66).

Transcriptional regulation of FA biosynthesis is controlled in a coordinated and positive manner by sterol regulatory element binding proteins (SREBPs) and by liver X receptors (LXR). LXR induces the expression of SREBP1, and both together participate in the upregulation of FA synthetic genes, especially ACC and FAS (67). However, in macrophages, these transcription factors have also other roles and fates. LXR has an anti-inflammatory role, antagonizing NF-κB action on inflammatory genes during TLR4 activation (68, 69). By participating in the upregulation of the plasma membrane cholesterol transporter ABCA1, LXR contributes to alter membrane cholesterol homeostasis, inhibiting the recruitment of the TLR adaptors MyD88 and TRAF6, and blocking in this way the activation of MAPK and NF-κB during TLR4 stimulation (70). In contrast, SREBP1 regulates the levels of some inflammasome receptors participating in IL1β production and inflammation development during LPS activation (71). Interestingly, it has also been described that SREBP1 activity exhibits a late increase during TLR4 challenge, thereby participating in the expression of genes that regulate the synthesis of anti-inflammatory PUFAs (72). In this manner, SREBP1 may participate in the resolution of inflammation.

In contrast with M1 macrophages, TAG uptake and FA release through lysosomal hydrolysis and oxidation appear to be essential for M2 macrophages (73, 74) (**Figure 2**). There is some controversy regarding the importance of FAO for M2 polarization. The first suggestion in support of this idea was a study using rather high doses of the CPT1 inhibitor etomoxir (≥ 50 µM) (75). Subsequent studies in human and mouse macrophages failed to confirm an effect of etomoxir on IL4 responses (33, 37, 76). The controversy was resolved by a study demonstrating that low concentrations of etomoxir (≤ 3 µM), while effectively inhibiting CPT-1, did not influence IL4 polarization (77). Moreover, high doses of etomoxir (>100 µM) deplete free intracellular CoA and IL4 driven polarization, even in the absence of CPT-1 or CPT-2. The effect is rescued by exogenous CoA treatment (77). While this study demonstrates that CPT-1 is dispensable for macrophage polarization to M2 and highlights the importance of CoA availability, future research using genetic approaches should shed more light on the importance of FAO in M2 polarization (36).

Another distinctive feature of IL4-treated macrophages is the up-regulation of CD36 by PPARγ, a signaling receptor and FA transporter that participates in macrophage alternative activation (73). CD36 plays a role in the uptake of LDL and VLDL particles, whose TAG molecules are metabolized by lysosomal acid lipases to produce free FA. These FA are sent to the mitochondria for β–oxidation, thereby enhancing OXPHOS in the M2 macrophages. In this way, CD36 influences the expression of M2 markers during IL-4 responses (73).

Interestingly, given that CD36 has multiple ligands, the receptor may support both anti- or pro-inflammatory macrophage activation depending on the environment, the ligands and the coreceptors used for signaling. For example, it has been described that in atherosclerosis, oxidized low-density lipoproteins (oxLDLs) in the intima of the arteries bind to CD36 with high affinity, activating signaling cascades that help during lipoprotein internalization and foam cell formation (78, 79). oxLDLs induce the expression of FA transport proteins (FABP4, ACSL1, CPT1, CPT2) that sequentially carry exogenous FA into the mitochondrial matrix. Contrary to the alternative activation, the oxLDL/CD36 axis reduces OXPHOS and switches metabolism to glycolysis, causing increased accumulation of free FA in the mitochondria and a parallel mitochondrial ROS production. Mitochondrial ROS activates the NF-κB pathway and promotes pro-inflammatory macrophage activation (80, 81). In this scenario, CD36 also participates in the activation of the inflammasome and IL1β production by facilitating the accumulation of cholesterol crystals that promote lysosomal disruption (82).

Microbiota-derived short-chain fatty acids (SCFAs), including acetate, propionate, and butyrate, have garnered significant attention for their impact on macrophage physiology. These metabolites, produced during the fermentation of dietary fibers by gut microbiota, play crucial roles in regulating immune responses and inflammation. For instance, butyrate has been shown to reduce the development of IFNγ generating cells while promoting the development of regulatory T cells, thereby modulating immune responses (83). Furthermore, SCFAs influence macrophage functions by promoting phagosome-lysosome fusion, increasing reactive oxygen species production, and balancing cytokine responses. These effects are particularly relevant in the context of pulmonary fungal infections, where SCFAs have been explored for their therapeutic potential (84). Additionally, SCFAs have been linked to a decrease in allergic inflammation in asthma, highlighting their role in modulating immune responses (85–87).

### 2.3 Oxylipins regulate macrophage polarization

**Figure 3:**
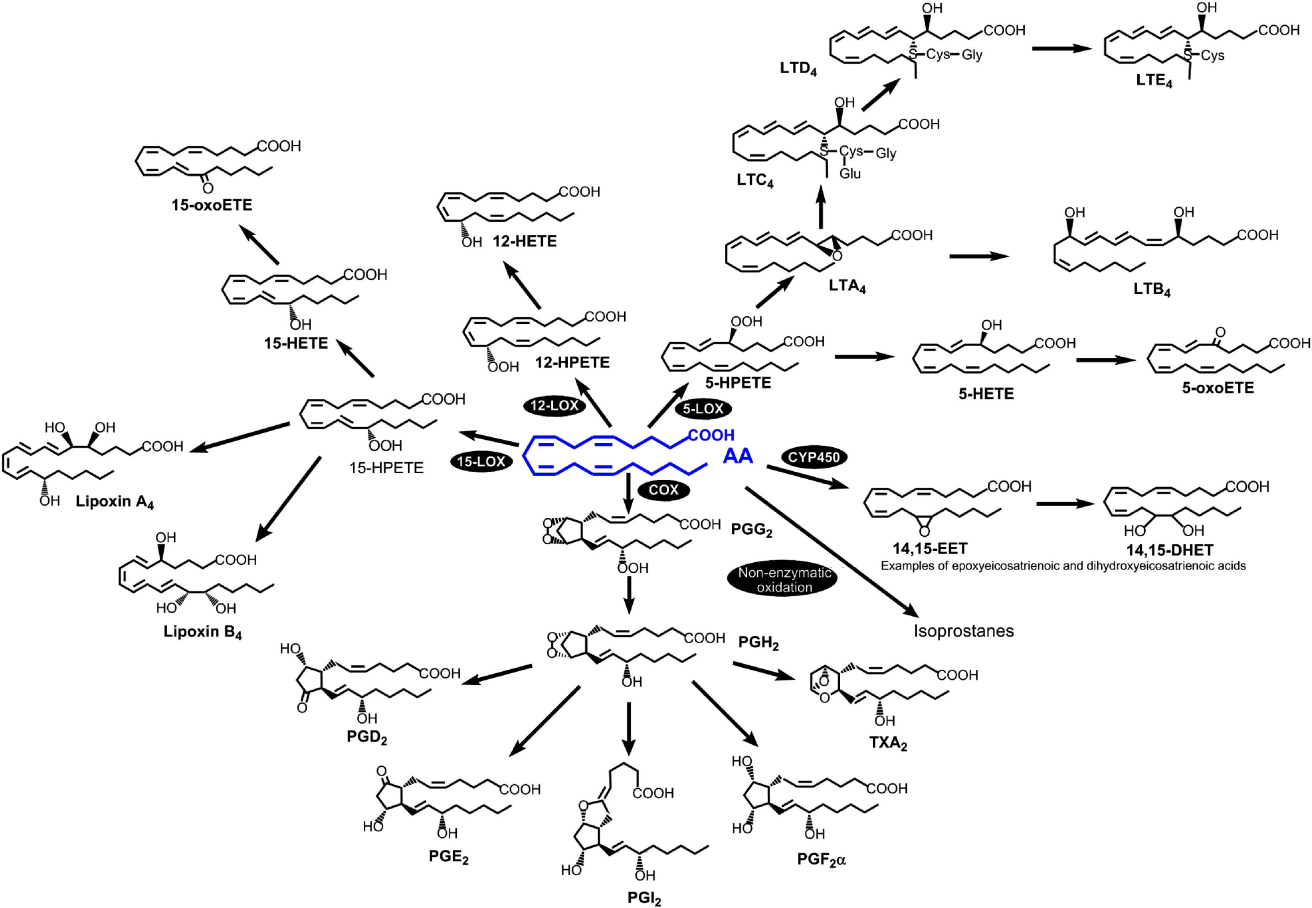
The AA-derived eicosanoid family. The four pathways for AA metabolism into eicosanoids are as follows: cyclooxygenase, lipoxygenase, cytochrome-P450 and non-enzymatic oxidation reactions. Reprinted with permission from ref. 88.

The oxylipins, including the AA-derived eicosanoids, are crucial endogenous mediators in physiological and pathological processes, synthesized by different cells but especially inflammatory cells. They are generated from PUFAs by various enzymatic pathways and include the prostaglandins (PG) and thromboxane (TX) (COX pathway), leukotrienes (LTs), lipoxins (LX) and hydroxyeicosatetraenoic acids (HETEs) (LOX pathway), and epoxyeicosatrienoic acids (ETEs) (via cytochrome P450). A fourth pathway, nonenzymatic, generates the isoprostanes (88, 89) (**Figure 3**).

It is widely known that the eicosanoids play a key role in inflammation, and are linked to cardiovascular diseases, metabolic syndrome, and cancer (89, 90). Thus, the identification of eicosanoid biomarkers and/or therapeutic targets are prominent areas of research. Even before the introduction of the concept of macrophage polarization, different laboratories showed how proinflammatory stimuli induce the synthesis or repression of enzymes that mediate eicosanoid metabolism, and produce specific profiles of eicosanoids (91). Gupta and co-workers (92) generated a comprehensive quantitative lipidomic data set of eicosanoids produced upon stimulation with the TLR4 ligand, Kdo2 lipid A (KLA) in RAW264 macrophage-like cells. Subsequently, Norris et al. (93) compared quantitative temporal differences in eicosanoid levels with the expression of enzymatic transcripts after TLR4 stimulation with KLA in four different types of macrophages (resident or thioglycollate-elicited peritoneal macrophages, bone marrow-derived macrophages, and RAW246.7 macrophage-like cells). All macrophage phenotypes produced primarily COX metabolites, with notable variations in PGI_2_ synthase expression and PGI_2_ production among them (93). Studies in human macrophages under multiple polarization conditions confirmed that the COX products PGE_2_ and PGD_2_ are the major metabolites produced under all polarization states; however, no changes were detected in the levels of these metabolites among the different conditions (94). Strikingly however, M1 macrophages displayed a remarkable synthesis of TXB_2_ which was not found in the other polarization phenotypes (94).

From another perspective, Lukic and colleagues (95) analyzed inflammatory lipid mediator profiles in macrophage phenotypes before full activation, using GM-CSF or M-CSF to prime cells towards M1 or M2 phenotypes. They considered this scenario because this is likely reflecting the state of the cell when pathogens are encountered. In resting conditions, both phenotypes released pro-resolving lipid mediators. Upon bacterial stimulation, M-CSF macrophages shifted towards proinflammatory eicosanoids, including increased 5-LOX products. However, GM-CSF cells exhibited higher 5-LOX expression, forming consequently high amounts of 5-HETE. This suggests pre-existing pathway preferences before full M1/M2 activation that influence the inflammatory response (95). Also working with pathogens, Werz and colleagues showed that human macrophages respond uniquely to bacteria (96). M1 macrophages, activated by *E. coli* and *S. aureus*, predominantly produce proinflammatory eicosanoids such as LTB_4_ and PGE_2_. In contrast, M2 macrophages respond to these pathogens by generating specialized pro-resolving mediators. This distinction in responses underscores the diverse roles of M1 and M2 macrophages in inflammatory or pro-resolving contexts during infections (96).

More recently, another study determined eicosanoid formation during macrophage polarization, and its subsequent role in inflammation. Cui and co-workers (97) developed an acute inflammatory model in mice after LPS inoculation and studied the inflammatory cytokine secretion (TNFα and IL6) along with the phenotype changes of peritoneal macrophages over time. They observed an increase in eicosanoids from the COX pathway and a decrease in products from the LOX pathway in the macrophages after LPS administration. To further clarify the relationship between macrophage polarization and eicosanoid metabolism, they differentiated THP-1 cells into M1 and M2 phenotypes *in vitro* and analyzed their eicosanoid content. The results showed that M1 cells utilized AA in preference, while M2 cells preferred EPA as a substrate, suggesting a potential mechanism for the anti-inflammatory properties of M2 macrophages (97).

### 2.4 Phospholipase A_2_s and acyltransferases modulate lipid signaling and macrophage function

**Figure 4:**
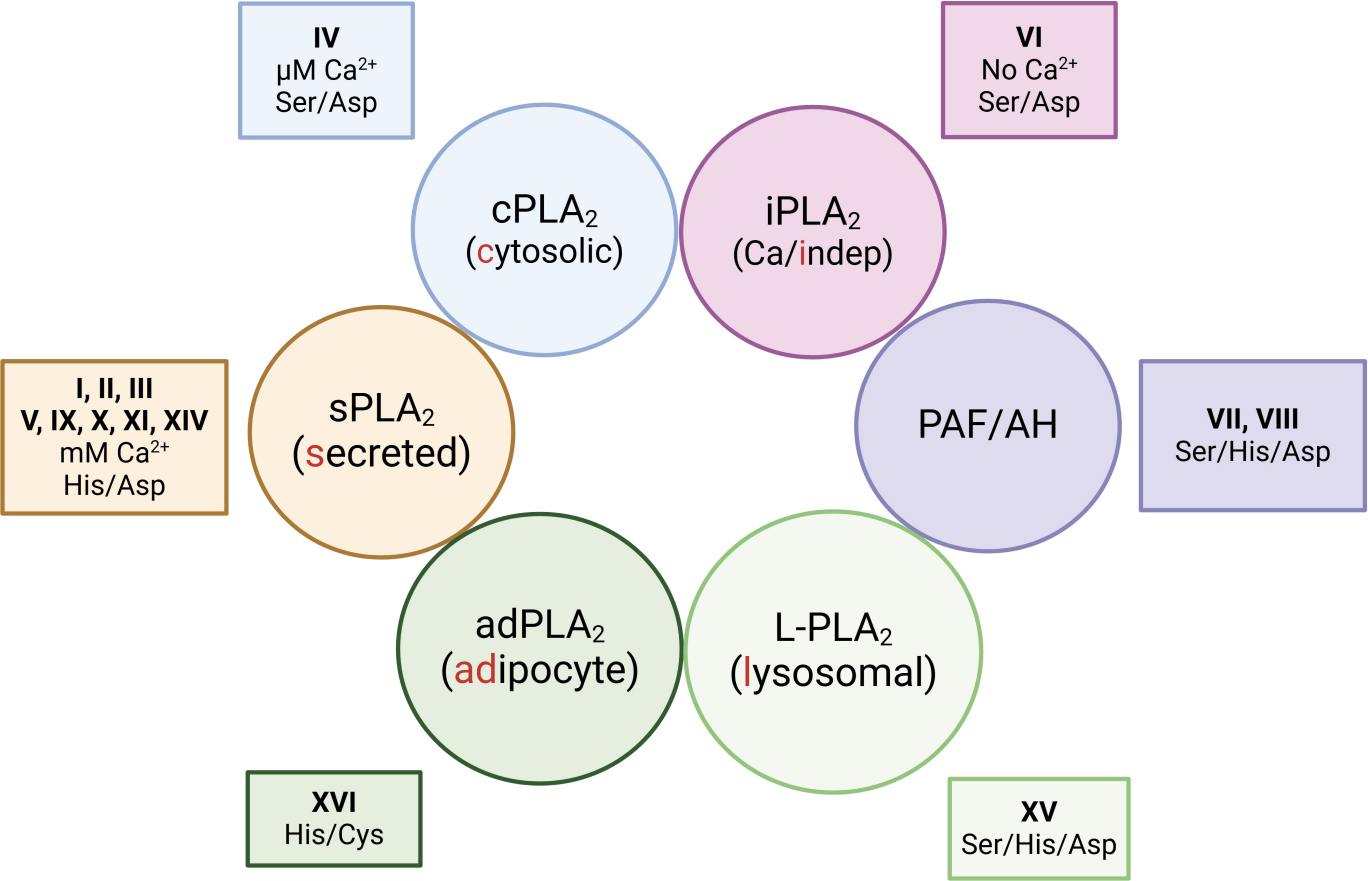
Functional classification of phospholipase A_2_s. The enzymes are grouped into 6 major families depending on biochemical commonalities i.e. whether they are secreted or reside in specific subcellular compartments; and whether they need Ca^2+^ for activity. The figure also includes the group numbers that each family belongs to, and the catalytic amino acid diads/triads of each family.

Phospholipases are the enzymes responsible for cleaving ester bonds within phospholipids. Their hydrolytic activity produces a variety of lipid products that play crucial roles in cellular signaling. Among the phospholipase superfamily, phospholipase A_2_s (PLA_2_s) are particularly important, as these are the enzymes that generate the free polyunsaturated fatty acids that serve as precursors for oxylipin biosynthesis (88, 98, 99). The PLA_2_ enzymes were initially classified into groups according to structural and sequence criteria. At the time of this writing, 16 groups have been described, with many of them including several subgroups (100, 101). However there is an alternative classification, frequently used, that categorizes the PLA_2_s on the basis of biochemical and functional similarities (100, 101). According to this classification, PLA_2_s are grouped into 6 major families, namely: (i) the sPLA_2_s or secreted enzymes; (ii) the cPLA_2_s, or calcium-dependent cytosolic enzymes; (iii) the iPLA_2_s, or calcium-independent enzymes; (iv) the platelet-activating factor acetyl hydrolases (PAF-AH); (v) the L-PLA_2_ or lysosomal enzymes and (vi) the adPLA_2_ or adipose tissue-specific enzymes (**Figure 4**).

**Figure 5:**
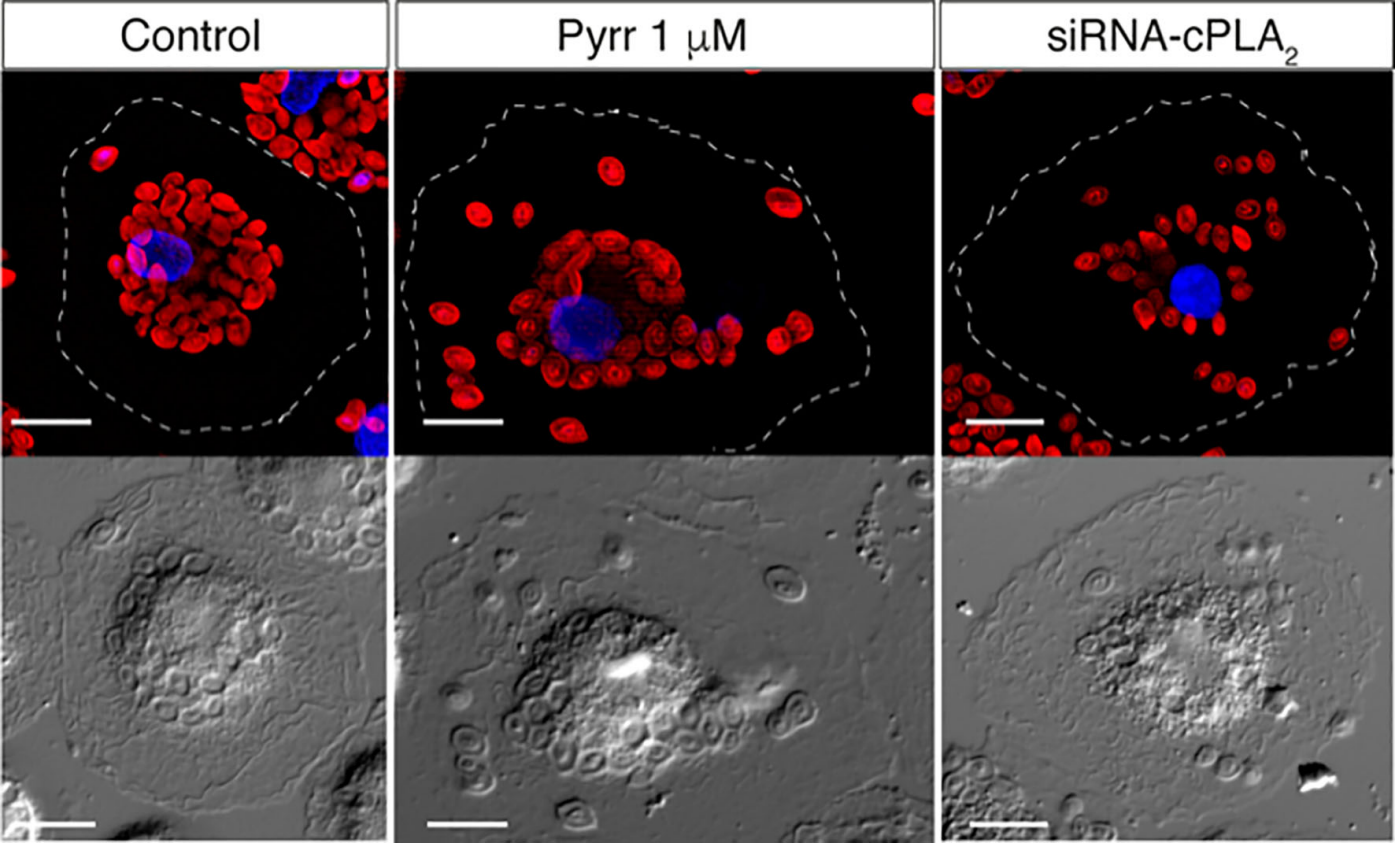
cPLA_2_α regulates phagosome internalization. Group IVA cytosolic phospholipase A_2_α (cPLA_2_α), the major enzyme controlling AA mobilization for production of eicosanoids during phagocytosis, is known to translocate to the phagosome membrane. The figure shows the internalization of IgG-opsonized Alexa Fluor594-conjugated zymosan particles by human monocyte-derived macrophages, as analyzed by epifluorescence microscopy. In control untreated cells, the particles are internalized deep inside the cell, concentrating in close proximity to the nucleus. In contrast, in cells treated with the inhibitor pyrrophenone (Pyrr) or cells depleted of the enzyme by specific siRNA silencing (siRNA-cPLA_2_α), a more disperse internalization pattern is observed, with a significant number of particles dispersed all over the cytoplasm, far away from the nucleus. Thus, cPLA_2_α actively modulates the internalization of the IgG-opsonized particles. Magnification, 10 µm. For further details, see refs. 106 and 107.

Since PLA_2_s usually display cellular expression specificities and defined substrate preferences, it has been long speculated that differentially polarized macrophages could exhibit select PLA_2_ profiles and, therefore, generate specific lipidomic signatures for each polarized activation state. Particularly important in this regard would be the expression profiles of the group IVA calcium-dependent cytosolic phospholipase A_2_ (cPLA_2_α), and the sPLA_2_s belonging to groups IIA, V, and X, as these are the enzymes most commonly implicated in regulating AA mobilization and attendant eicosanoid biosynthesis (100–103). Of note, in addition to regulating lipid signaling, cPLA_2_α is also critical to sustain another major macrophage function, i.e. phagocytosis. This enzyme is well documented to translocate to the phagosome during phagocytosis (104–106). cPLA_2_α seems essential for the cell to effectively internalize the ingested particles through mechanisms involving both its catalytic activity and the specific interaction of its C2 and polyphosphoinositide-binding domains with the phagosome membrane (107–109) (**Figure 5**).

In studies with murine peritoneal and bone marrow-derived macrophages, Ashley and co-workers assessed the expression of a number of PLA_2_ enzymes, namely cPLA_2_α and the calcium-independent enzymes iPLA_2_β (group VIA PLA_2_) and iPLA_2_γ (group VIC PLA_2_) (110). Of these, only cPLA_2_α was found to slightly increase upon activation with either LPS plus IFNγ (M1 conditions) or IL4 (M2 conditions). Interestingly however, analyses of macrophages from iPLA_2_β^–/–^ k.o. mice suggested the involvement of this enzyme, not cPLA_2_α, in macrophage polarization. Stimulation of iPLA_2_β-deficient macrophages with IL4 resulted in the increased expression of M2 markers, as compared with macrophages from wild type animals. These findings suggested that the absence of iPLA_2_β drives the macrophages to an M2 phenotype, while its presence favors an M1 phenotype (110). Further studies from the same group concluded that iPLA_2_β also participates in the regulation of the production of pro-inflammarory oxylipins by the macrophages (111).

In contrast with the above data, Klement and co-workers (112) reported that persistent treatment of mice with LPS markedly increases the mRNA expression level of pla2g4a, the gene coding for cPLA_2_α. Moreover, a trend and significant further upregulation of pla2g4a was observed in male and female iPLA_2_β^–/–^ k.o. mice, respectively, suggesting that the lack of iPLA_2_β up-regulates cPLA_2_α, this resulting in elevated production of eicosanoids (112). The same work also showed that specific iPLA_2_β deficiency in myeloid cells leads to the promotion of adaptive autoimmune and LPS-innate inflammatory responses associated with an activation of MIP-1α/CCL3 (112). Thus the absence of iPLA_2_β exacerbating inflammation suggests that a major role for iPLA_2_β in macrophage physiology is to regulate lipid signaling pathways that orchestrate the generation of protective, anti-inflammatory M2-type responses (113). Consistent with this view, iPLA_2_β has repeatedly been observed to function as the major PLA_2_ effecting the mobilization of anti-inflammatory FAs such as adrenic acid (114, 115), DHA (116–118), and palmitoleic acid (119, 120). The key role that iPLA_2_β appears to play in clearing oxidized membrane phospholipids (121, 122), is also in agreement with an anti-inflammatory role for this enzyme in macrophages.

Among the PLA_2_ family of enzymes, the secreted PLA_2_s (sPLA_2_) constitute the largest class, with at least 11 different enzymes being expressed by mammalian cells. In particular, sPLA_2_ enzymes are abundantly present in innate immune cells, where they play specific roles in regulating a wide variety of innate immune functions, ranging from the production of bioactive oxylipins to membrane rearrangement or the degradation of the membrane of invading pathogens or extracellular vesicles (123). Among the sPLA_2_s, the group V enzyme (sPLA_2_-V) represents a very interesting case because of its bi-faceted role in immunity (103, 124, 125), i.e. the enzyme may play both pro- and anti-inflammatory roles depending on conditions, cell type, and species. This enzyme hydrolyzes phospholipid-bound oleic and linoleic acid residues in preference to AA residues (126, 127), suggesting varied roles for this enzyme in addition to or independent of its role in the eicosanoid metabolic cascade (123, 128). As regards to the latter, studies utilizing sPLA_2_-V-deficient mice have conclusively demonstrated the involvement of this enzyme in regulating AA mobilization and the attendant eicosanoid generation of macrophages during phagocytosis of zymosan particles (129). Subsequent studies demonstrated as well the involvement of sPLA_2_-V on stimulation of the macrophages via TLR1/2, TLR2, TLR3, TLR4, TLR6/2 and TLR7, but not TLR5 or TLR9 (130–132). It is interesting to note in this regard that clear variations in the lipidome of macrophages stimulated via different receptors have been described (133). This suggests that, depending on the receptor involved, different phospholipases may be implicated in shaping the cellular lipidome and thus in guiding the macrophages to their effector functions. During TLR4 stimulation, sPLA_2_-V is thought to amplify the action of cPLA_2_α, which is the key enzyme in effecting the release of AA (134). While the molecular details governing cross-talk between the two PLA_2_s remain to be fully clarified, it has been shown that sPLA_2_-V regulates cPLA_2_α phosphorylation (132, 135). Alternatively, sPLA_2_-V-catalyzed phospholipid hydrolysis may produce lipid metabolite(s) that directly regulate(s) the activity of the cPLA_2_α, or vice versa (136–138).

The involvement of sPLA_2_-V in regulating the production of AA-derived proinflammatory lipid mediators suggests detrimental roles for this enzyme under certain circumstances. However, comprehensive analyses of PLA_2_ expression in macrophages from different types and species has revealed that sPLA_2_-V is very strongly induced by stimuli that promote polarization to an anti-inflammatory M2 phenotype, including IL4, IL13, IL10, and M-CSF (139–141). Increased sPLA_2_-V expression in M2 polarized human macrophages correlates with the greater phagocytic capacity that these cells exhibit, suggesting that sPLA_2_-V-regulated lipid changes may be crucial for the execution of this response (141–143). In keeping with this view, lipidomic analyses of human macrophages identified the ethanolamine lysoplasmalogens as sPLA_2_-V-derived metabolites that effectively restore phagocytosis in sPLA_2_-V-deficient cells (141, 144). More recent work using bone marrow-derived macrophages from sPLA_2_-V deficient mice, confirmed the essential role that this enzyme plays in phagocytosis and in the activation of select lipid pathways during polarization towards an M2 phenotype, in particular those involving the hydrolysis of ethanolamine phospholipids (125). Importantly, the latter study also confirmed the role of sPLA_2_-V in regulating AA mobilization and eicosanoid production regardless of the polarization activation regime, thus stressing again the bi-faceted role of sPLA_2_-V, modulating both pro- and anti-inflammatory actions. Under both polarization regimes, PGE_2_ and PGD_2_, were reduced in macrophages lacking sPLA_2_-V (125).

PUFAs are not typically incorporated into phospholipids during *de novo* biosynthesis in the endoplasmic reticulum; instead, they are integrated at a later stage via a deacylation/reacylation cycle known as the Lands cycle (145–148). In this cycle, PLA_2_ enzymes, particularly the calcium-independent ones (149–151), cleave existing phospholipids to generate 2-lysophospholipids, which are then rapidly reacylated with PUFAs by CoA-dependent lysophospholipid acyltransferases (145–148). These latter enzymes thus play a crucial role in lipid remodeling and the generation of bioactive lipids. By modulating the composition of cellular membranes, the CoA-dependent acyltransferases influence various cellular functions, including membrane fluidity, curvature, and signaling pathways. In macrophages and other innate immune cells, these enzymes are crucial for maintaining lipid homeostasis and facilitating appropriate responses to environmental stimuli (152–154). The activity and expression of specific acyltransferases have significant implications for macrophage biology. For instance, lysophosphatidylcholine acyltransferase 2 (LPCAT2) is known to co-localize with cyclooxygenase-2 (COX-2) in lipid droplets within macrophages (155). This association suggests a role for LPCAT2 in the production of inflammatory mediators, as lipid droplets are described as sites for the synthesis of eicosanoids (156). Moreover, LPCAT2 is required for macrophage cytokine gene expression and release in response to TLR4 and TLR2 ligand stimulation, but not for TLR-independent stimuli, indicating its selective involvement in pathogen-induced inflammatory responses (157). Additionally, the regulation of lysophospholipid levels by acyltransferases can impact macrophage activation states. Elevated levels of lysophospholipids, such as lysophosphatidylcholine (LPC), have been associated with pro-inflammatory macrophage activation (158, 159). By controlling the reacylation of LPC, enzymes like LPCAT3 help modulate the inflammatory status of innate immune cells (154). Characterization of human LPCAT3 has shown its robust activity in esterifying lysophosphatidylcholine with PUFA, especially AA (160, 161), thereby limiting the abundance of bioactive lysophospholipids that can act as signaling molecules in inflammation.

### 2.5 Glycerophospholipids and sphingolipids in the dynamics of macrophage polarization

In addition to liberating free FAs, PLA_2_ activation leads to the production of lysophospholipids, many of which are biologically active (162). Lysophospholipids play a crucial role in macrophage biology, influencing various aspects of immune function, inflammation, and cellular signaling. Macrophages utilize these lipids to regulate their activation states, migration, and cytokine production in response to infections or tissue injury. Through interactions with specific G-protein-coupled receptors lysophospholipids modulate intracellular signaling cascades that influence pro-inflammatory or anti-inflammatory responses depending on the context (163, 164). As indicated in the preceding section, ethanolamine lysoplasmalogens appear to play a crucial role in regulating the elevated phagocytic response exhibited by M2 macrophages (141, 144). LPC has been implicated in promoting inflammatory responses by stimulating TLR pathways and, as a consequence, inducing COX-2 and the production of cytokines such as TNFα and IL6 (131, 165). On the other hand, lysophosphatidic acid signaling can enhance macrophage survival and migration, facilitating tissue remodeling and resolution of inflammation (163). These differential effects highlight the importance of lysophospholipid metabolism in fine-tuning immune responses during infections, autoimmune diseases, and tissue repair. Additionally, lysophospholipids may contribute to macrophage-driven pathologies, such as atherosclerosis and chronic inflammatory diseases. Oxidized phospholipids, including oxidized LPC, are known to accumulate in atherosclerotic plaques and modulate macrophage foam cell formation, promoting disease progression (159, 166).

During pro-inflammatory activation of macrophages, the levels of many glycerophospholipid species show significant increases (133, 167, 168). Dennis and colleagues first conducted analyses of lipid molecular species using quantitative mass spectrometry to delineate the macrophage lipidome following stimulation of RAW264.7 macrophage-like cells with Kdo2-lipid A, the active component of LPS (167). The data stressed that activation of the innate immune system by inflammatory mediators leads to alterations across a wide range of mammalian lipid categories (167). In general agreement with these data, Lee and collaborators (168) also characterized the lipid profile of RAW264.7 cells stimulated with different concentrations of LPS. This work showed that 11 classes of lipids, including TAG, DAG, ChE, PE, PS, PI, PA, LPC, LysoPE, Cer, and dCer, were increased, and that three classes, cholesterol, PC, and LysoPA, were decreased in an LPS concentration-dependent manner. Similar studies utilizing human macrophages also demonstrated profound changes in the cellular phospholipidome on stimulation with innate immune stimuli (94). In these studies, a lipidomic analysis of 4 macrophage phenotypes (M1, M2a, M2c and controls) was performed, and a principal component analysis revealed substantial changes in the lipid profile of all activation states. M1 macrophages were the furthest apart in PC1, while both M2 groups were distinct from controls and M1, yet closely clustered in PC1 and PC2. However, in PC3, substantial differences between M2a and M2c were recognized. This study also reported the phospholipid composition of each macrophage phenotype, finding interesting variations in each phospholipid class. Species such as PE(34:1), PI(34:2) and PI(38:5) showed slight increases (differentiated cells compared to controls) in contrast to PE(38:4) or PI(38:4), which decreased (94). Decreases in these latter species along with other species such as PC(36:4), PC(38:4) and PC(40:5) have also been documented in other studies, and are indicative of stimulus-induced AA mobilization (169–173). However, in other cases, the phospholipid alterations appear to be driven by an upregulation of *de novo* pathways for lipid synthesis. In support of this, the removal of key enzymes involved in this process, such as acetyl-CoA carboxylase, attenuates glycerophospholipid enrichment (66). Additionally, the levels of glycerolipid synthesis intermediates—particularly saturated and monounsaturated species of phosphatidic acid and its derivative diacylglycerol—increase during TLR4 activation (167), reinforcing further the role of the *de novo* synthesis in this context.

**Figure 6:**
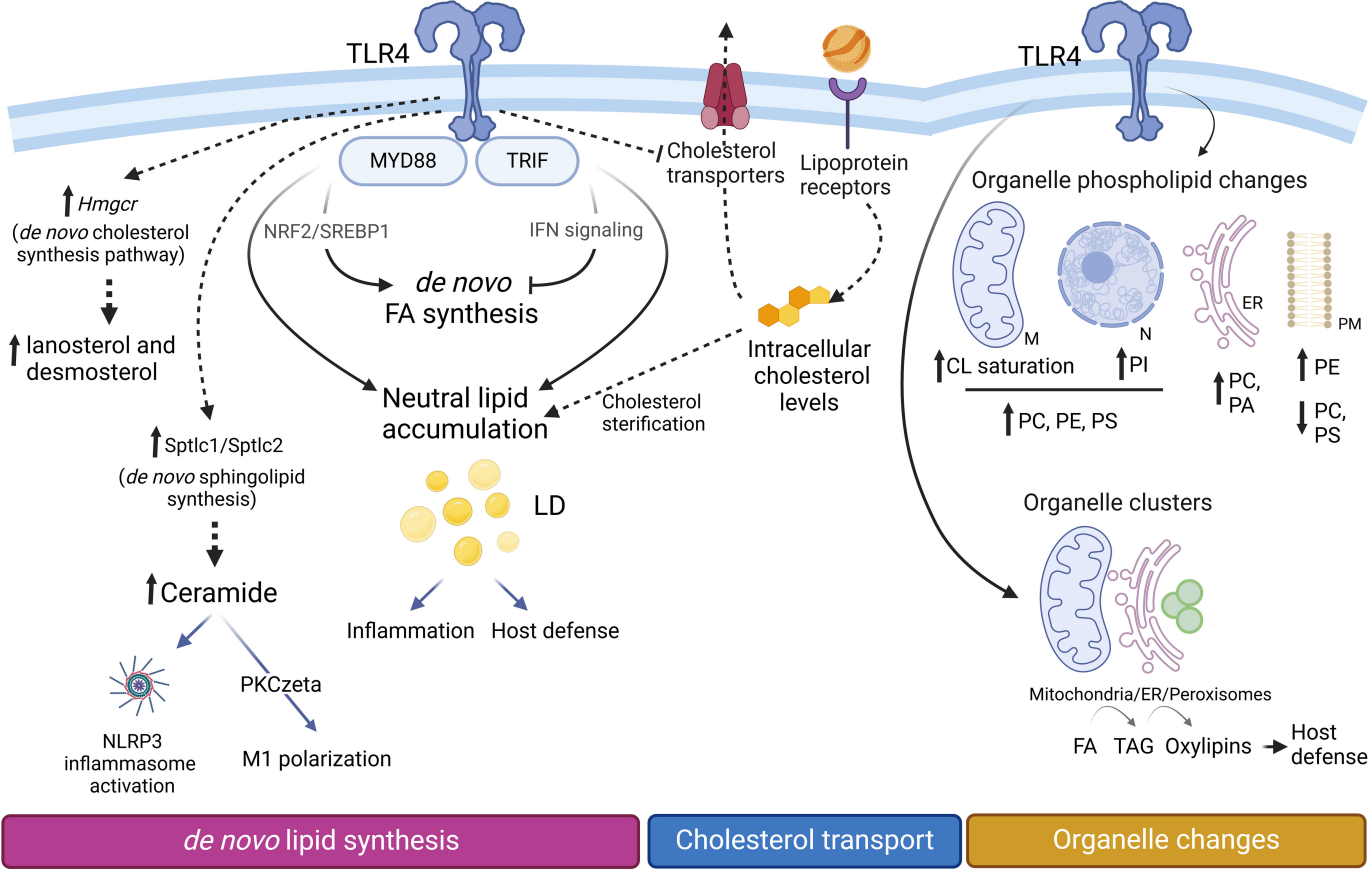
Lipid changes during M1 polarization. Proinflammatory macrophages accumulate triacylglycerol (TAG) and cholesterol esters (CE) within lipid droplets (LD), increasing their number and size. TAG synthesis, critical for LD formation, supports inflammation by providing precursors for lipid mediators and contributes to host defense mechanisms. Upregulation of *Hmgcr* facilitates the production of cholesterol biosynthetic intermediates such as lanosterol and desmosterol. However, cellular levels of cholesterol and CE are primarily regulated through lipoprotein internalization and alterations in the expression of cholesterol transporters. Increased expression levels of *Sptlc1/Sptlc2* promote an elevation of *de novo* sphingolipid synthesis. The glycerophospholipid composition of cellular organelles undergoes significant changes. In mitochondria, cardiolipins (CL) become more saturated, while PC, PE, and PS increase in both mitochondria (M) and the nucleus (N). The endoplasmic reticulum (ER) becomes enriched in phosphatidic acid (PA), whereas the plasma membrane (PM) experiences an increase in PE and a concomitant decrease in PC and PS levels. Organelle clustering facilitates the biosynthetic flow of FA (from mitochondria) to TAG (in the ER) and oxylipins (in peroxisomes), enabling coordinated lipid metabolism and inflammatory responses.

Subcellular lipidomic analyses have suggested that, during activation of macrophages via TLR4, glycerophospholipids changes occur not only in the plasma membrane but also in intracellular membranes (**Figure 6**). After separation of different cellular fractions by centrifugation, Andreyev and co-workers (174) noted that the nuclear and mitochondrial fractions increase their enrichment in PC, PE, and PS; the endoplasmic reticulum fraction increases its content of PC and PA, and the plasma membrane fraction increases its content of PE while decreasing PC and PS. Interestingly, species of PI primarily increase in the nuclear fraction. Therefore, the nucleus and mitochondria are the organelles that show the greatest increase in glycerolipid content during the pro-inflammatory activation of macrophages (174). These changes are likely related with the finding that specific intracellular sites exist for the different signal-activated phospholipases to mediate phospholipid turnover and signaling (99, 175, 176).

Cardiolipins are glycerophospholipids that reside specifically in the mitochondrial inner membrane. These complex lipids, containing four FAs, undergo extensive turnover after macrophage activation, leading to cardiolipin species with fewer unsaturations and shorter chains (174). Due to the important role of these lipids in the maintenance of cristae morphology and stability and function of ETC complexes, cardiolipin remodeling is suggested to participate in the modulation of respiratory chain function during inflammatory reprograming of macrophages (177, 178).

Sphingolipids are crucial regulators of macrophage function, particularly during inflammatory responses and polarization. Molecules such as sphingosine and ceramide and their phosphorylated derivatives were originally described as having opposing roles. Phosphorylated sphingolipids, including ceramide-1-phosphate and sphingosine-1-phosphate, support cell survival by promoting proliferation, inflammation, and motility. Conversely, their non-phosphorylated counterparts, ceramide and sphingosine, induce apoptosis, cell cycle arrest and senescence, collectively forming the “sphingolipid rheostat” (179). Similarly, ceramide and sphingosine-1-phosphate exhibit distinct roles in macrophage polarization (180). Ceramide drives pro-inflammatory M1 polarization by activating pathways such as NLRP3, leading to the production of IL1β (181). For example, C2-ceramide reprograms tumor-associated macrophages toward an M1 phenotype through protein kinase Cζ-dependent mechanisms, enhancing pro-inflammatory cytokine secretion and improving CD8^+^ T cell cytotoxicity within the tumor microenvironment. These findings underscore the contribution of ceramide to antitumor immunity (182, 183). In contrast, the influence of sphingosine 1-phosphate on macrophage functionality is more nuanced. It signals through five G protein-coupled receptors (S1PR1–S1PR5), each linked to distinct pathways. Depending on the receptor involved and the microenvironment, sphingosine 1-phosphate can promote either pro-inflammatory or anti-inflammatory responses, emphasizing its dual role in immune regulation (184, 185).

Macrophage activation through TLR4 also leads to acute alterations in sphingomyelin levels via sphingomyelin synthase (186, 187). At longer times, an increase in palmitoyl-CoA and the expression of serine palmitoyltransferase, the rate-limiting enzyme in sphingolipid synthesis, drives the *de novo* production of sphingolipids (167). This synthesis contributes to the lipid remodeling that facilitates adaptation of the macrophages to their microenvironment and phenotypic shifts. Hence, targeting these pathways may offer promising therapeutic potential to redirect macrophage polarization, either to mitigate chronic inflammatory diseases or to bolster antitumor immunity.

### 2.6 Neutral lipids: Triacylglycerol and cholesterol esters

Triacylglycerol (TAG), an energy-storing neutral lipid, significantly increases its levels under proinflammatory conditions, and plays important roles in regulating inflammation (133, 167, 188). This lipid, along with cholesterol esters, is stored in lipid droplets, resulting in a notable increase in both the number and size of these organelles in proinflammatory macrophages (**Figure 6**) (133, 188, 189). Interestingly, the inhibition of TAG synthesis during LPS activation appears to prevent not only lipid droplet development but also the production of inflammatory mediators (188). Although the primary function of lipid droplets has traditionally been described as energy storage for ATP production via mitochondrial FAO, they are now recognized as hubs for antibacterial protein accumulation and reservoirs of precursors for proinflammatory lipids (188–190). Thus increased TAG synthesis and its accumulation in lipid droplets may support host defense (191), but it can also exacerbate atherosclerosis development during infections and inflammatory diseases (192).

Recent studies using a multi-spectral organelle imaging approach that enables the simultaneous visualization and behavioral analysis of several organelles, have shown that during M1 polarization, a flux of FA exists within clusters of endoplasmic reticulum and mitochondria to supply the necessary FA for lipid droplet growth. The subsequent recruitment of peroxisomes to these clusters supports the hydrolysis of TAG to release PUFA which may putatively mediate lipid signaling via oxylipin formation (190). The molecular mechanism for TAG hydrolysis and effector(s) involved in FA release under these conditions remain to be clarified, as cPLA_2_α, an enzyme that does not hydrolyze TAG, is well established as the essential enzyme for eicosanoid formation in major immunoinflammatory cells such as macrophages and mast cells (193–196). Conversely, cPLA_2_α is known to regulate lipid droplet formation in human phagocytes (197, 198).

Other studies, using a combination of shotgun lipidomics and stable-isotope tracing, revealed how macrophages change their neutral lipid profile when activated through TLRs and also during polarization to M1 (LPS plus IFNγ) (133). Macrophages activated only through TLR4 or activated with LPS plus IFNγ accumulate lipids differently. Combining LPS with IFNγ enhances the reprograming of the lipidome promoted by LPS alone, further increasing the accumulation of cholesterol esters and TAG. Although both MyD88 and TRIF adaptor proteins contribute to neutral lipid accumulation during TLR4 activation, they have opposing effects on *de novo* FA synthesis. MyD88 signaling promotes *de novo* synthesis of long chain FA, particularly monounsaturated FA such as oleic acid, which depends on NRF2 and SREBP1 transcriptional activities. In contrast, TRIF signaling, which is involved in the upregulation of IFN, opposes MyD88 in FA synthesis through autocrine type I interferon signaling. However, both MyD88 and TRIF participate in the accumulation of neutral lipids during TLR4 activation, which possibly reflects different roles in lipid uptake and traffic (133, 199).

Proinflammatory activation of macrophages leads to elevated levels of intermediates of the *de novo* synthesis of cholesterol, including lanosterol and desmosterol. Lanosterol accumulation attenuates the activation of STAT1-STAT2 signaling pathways, resulting in decreased secretion of proinflammatory cytokines (200). Desmosterol accumulation suppresses inflammasome activation and modulates inflammatory responses in macrophages through pathways independent of LXR activation, highlighting the complexity of cholesterol metabolism in immune regulation (201). Likewise, other oxysterols such as 25-hydroxycholesterol and 24,25-epoxy-cholesterol are also elevated (167, 200, 202). These oxysterols influence macrophage polarization primarily through the activation of LXRs. When activated by oxysterols, LXRs promote the expression of genes responsible for cholesterol efflux and anti-inflammatory responses, thereby facilitating the polarization of macrophages toward an anti-inflammatory phenotype (203). In addition, these increases are accompanied by a significant upregulation of the expression of HMG-CoA reductase (Hmgcr), a limiting step in the pathway, and of cholesterol 25-hydroxylase (Cho25h), which participates in the production of 25-hydroxycholesterol (167). The most pronounced changes involve the up-regulation of sterol-O-acyl transferases -1 and -2, and the accumulation of cholesterol esters. Unexpectedly however, the latter changes appear to be driven by different processes. While the cholesterol biosynthesis pathway contributes to the accumulation of intermediates, the final concentration of cholesterol and its esters seems to be primarily regulated by the uptake of lipoproteins through membrane receptors (Ldlr, Cd36, Msr1, etc.), along with changes in the expression of transporters (Abca1, Abcg1) that export cholesterol (167) (**Figure 6**).

M2-polarized macrophages exhibit lower levels of both TAG and cholesterol esters compared to M1 macrophages. They also exhibit a markedly different behavior in response to an overload of exogenous free FA. While M1 macrophages primarily accumulate these FA in TAG, M2 macrophages accumulate them in glycerophospholipids and sphingophospholipids (204). This behavior, along with the differences between these two populations regarding the utilization of FA for energy, could explain the disparities in lipotoxicity that M1 and M2 macrophages exhibit in pathophysiological conditions such as obesity (205). Future investigation should shed light into how these different macrophage populations channel *de novo* fatty acids into different families of complex lipids.

## 3 Macrophage lipid metabolism in aging: Some considerations

**Figure 7:**
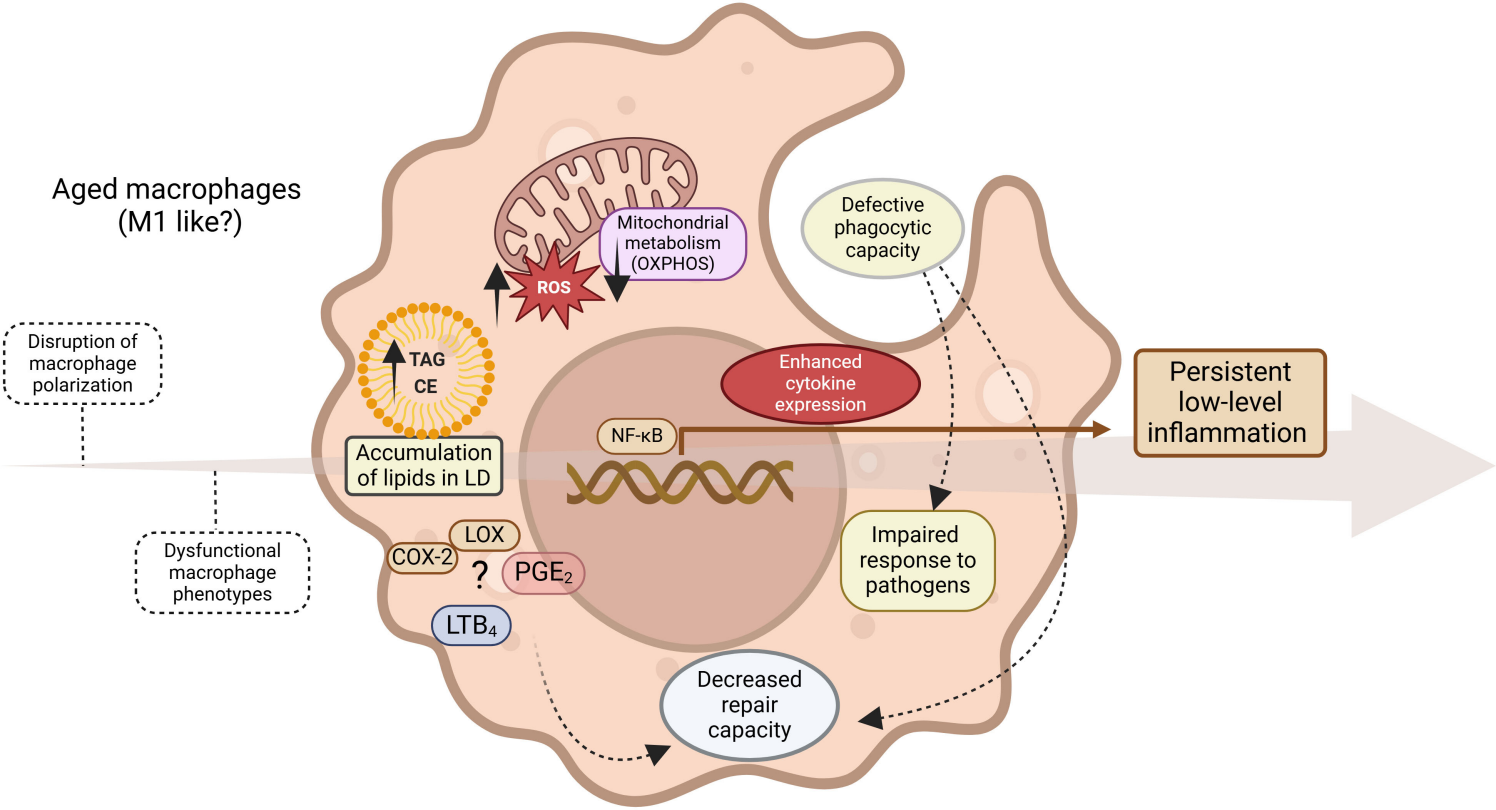
Inflammaging. Progressive evolution (arrow) of persistent non-infectious inflammation driven by multiple events, including dysregulation of lipid metabolism, results in a dysfunctional, predominantly proinflammatory macrophage phenotype with impaired immune functions.

Aging and age-related diseases are linked by fundamental mechanisms that often revolve around inflammation. As we age, a persistent, non-infectious, low-level inflammation state known as “inflammaging” emerges, playing a role in the development of age-related diseases (206–208) (**Figure 7**). Aging impairs macrophage polarization, leading to dysfunctional phenotypes that do not easily fit into the typical M1 or M2 categories. In general, tissue macrophages become more proinflammatory and less phagocytic with age. The altered tissue macrophages also contribute to organ deterioration in aging, as the decrease in cellular repair processes perpetuates damage at the level of nucleic acids and proteins.

Tissue resident macrophages adapt their metabolism to the microenvironment and the function that they have in each tissue (209, 210). In accordance with this, the changes that macrophages experience during aging are generally related to alterations in their metabolism triggered by local tissue perturbations (211), albeit distal perturbations such as changes in the gut microbiota may also contribute (212). Many tissue resident macrophages, especially alveolar macrophages, large peritoneal macrophages, Langerhans cells, Kupffer cells and splenic red pulp macrophages, are completely dependent on OXPHOS to survive, as deletion of OXPHOS genes reduces their viability (208). In this regard, aged tissue macrophages decrease their mitochondrial metabolism, especially through reduction of OXPHOS activity, increase ROS production and store large amounts of neutral lipids in lipid droplets (213–215).

In the aged brain, microglia (the brain macrophages) display accumulation of lipids in lipid droplets, defective phagocytosis, and increased ROS levels and proinflammatory cytokine production during LPS challenge (215). Lipid droplet-laden microglia display altered expression of multiple genes, including those related with the phagosomal maturation pathway, genes related with the synthesis of FA, and genes related with FA β-oxidation (215). These alterations seem to be dependent on lipid accumulation. Interestingly, lipid droplet-laden microglia displaying a dysfunctional phenotype are present in a mouse model of frontotemporal dementia (215). Other studies have shown that the prostaglandin receptor EP2 on myeloid cells participates in the suppression of bioenergetic metabolism, a process mediated by PGE_2_. Mechanistically, PGE_2_ drives the conversion of glucose to glycogen, with a concomitant reduction of glycolysis and OXPHOS and, consequently, ATP production. As a result, there is an increased proinflammatory immune response and cognitive decline of the aged animals. Importantly, inhibition of myeloid or peripheral EP2 prevents the cognitive decline (216). Finally, knock-in mouse models that express the human APOE4 allele, related with the late-onset of Alzheimer disease, have shown that the microglia of aged animals strongly express APOE. When subject to an inflammatory challenge, these cells respond by increasing glycolysis, Hif1a expression and reducing mitochondrial respiration, a phenotype that is characteristic of classically activated macrophages (217).

Recently, Schädel and collaborators used lipidomic profiling to demonstrate that aged peritoneal macrophages produce significantly decreased amounts of pro-inflammatory PGE_2_, LTB_4_, and specialized pro-resolving mediators such as maresin-1 and protectin DX (218). These declines were correlated with reduced expression of COX-1 and FLAP (5-lipoxygenase activating protein). This study is significant because it challenges the concept that aging solely increases the pro-inflammatory activation of macrophages (208–210). Rather, it is the disruption of lipid metabolism and signaling as a whole that affects immune functions.

Lipid metabolism is intricately linked to the development of cardiometabolic diseases, including atherosclerosis. As individuals age, dysregulation in lipid homeostasis contributes to the accumulation of lipids, inflammation, and vascular dysfunction, ultimately increasing the risk of a wide range of cardiovascular and metabolic disorders. Type 2 diabetes often coexists with coronary artery disease, contributing to heightened inflammatory infiltrates and enlarged necrotic cores, which worsen atherosclerosis. In this context, Bi and colleagues (219) showed that PGE_2_ regulates M2 polarization via the cyclic AMP-responsive element binding/brain-derived neurotrophic factor/tyrosine kinase receptor B pathway. Thus, *in situ* synthesis of PGE_2_ could be an attractive target to improve treatment strategies. Along the same lines, another significant complication is diabetic cardiomyopathy, characterized by cardiac inflammation and metabolic alteration. During this condition, altered FA metabolism drives macrophage polarization toward the pro-inflammatory M1 phenotype. Sreedhar et al. (220) demonstrated that the 14-3-3η protein plays a pathogenic role, as its silencing increases the expression of FAS and M1 cellular markers, and decreases M2 markers. In turn these data support a potential therapeutic target role for this cardiac protein in diabetic cardiomiopathy (220).

**Figure 8:**
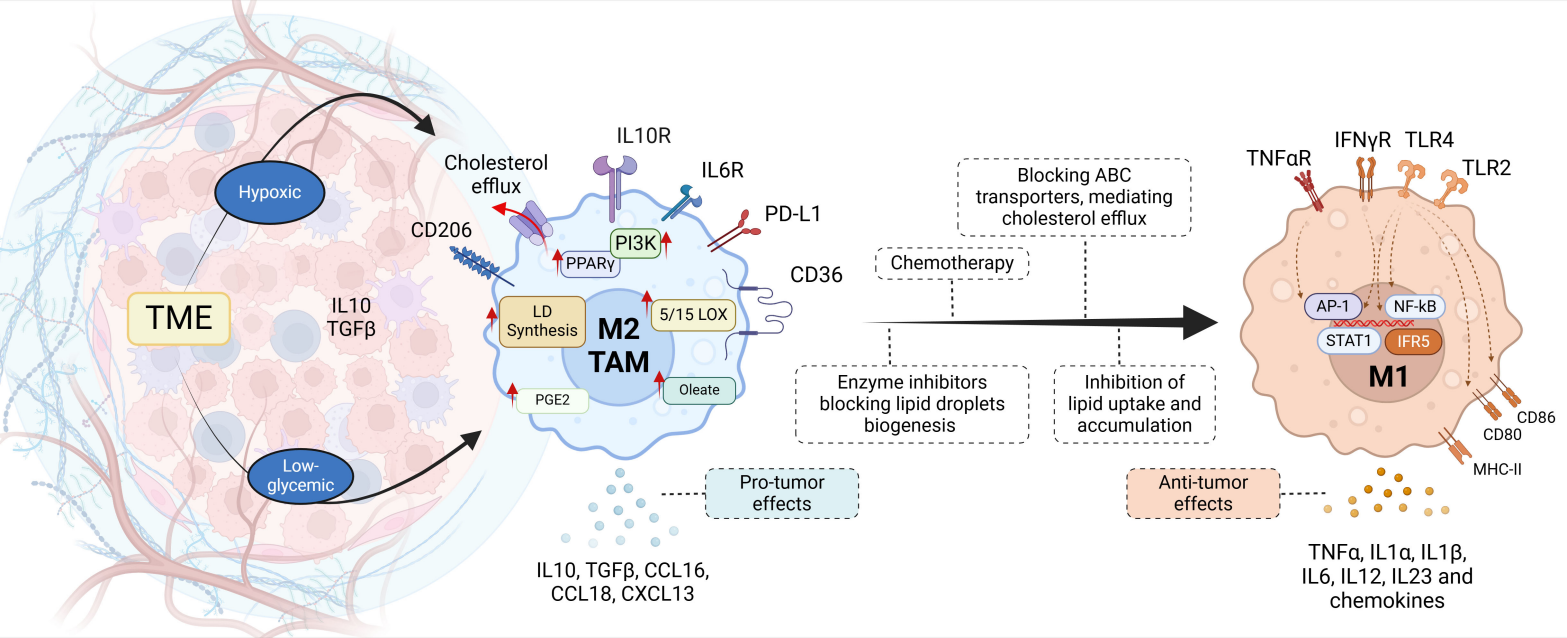
Tumor-associated macrophages. Recent therapeutic strategies focused on lipid metabolism aim to repolarize M2 macrophages in the tumor microenvironment (TME). Chemotherapy induces the production of ROS and the activation of HIF1α, reprogramming TAM metabolism to M1 with the release of pro-inflammatory mediators (cytokines and chemokines). M2 TAMs are characterized by exacerbated lipid synthesis pathways, mediated by the overexpression of PPARγ or PI 3-kinase (PI3K), leading to lipid accumulation. Thus the inhibition of key enzymes that mediate the synthesis of fatty acids, phospholipids, cholesterol, lipid droplets, or cholesterol efflux transporters, could represent therapeutic targets for pharmacological blockade.

Emerging evidence suggests that lipid metabolism also plays a pivotal role in age-related cancer progression, linking metabolic reprogramming to tumor development and immune dysfunction in aging tissues (221, 222). Tumor-associated macrophages (TAM), natural immune cells abundant in the tumor microenvironment, can be categorized into the anti-tumor M1 subtype and pro-tumor M2 subtype. Given the high plasticity of TAM, the shift from the M1 to M2 phenotype in the hypoxic and hypoglycemic tumor microenvironment accelerates cancer progression, closely tied to lipid metabolism. Since lipid metabolism reprogramming profoundly impacts TAM function, it holds potential as a promising therapeutic target for cancer (**Figure 8**). Key players of lipid metabolism in TAMs, including PPARγ, PI 3-kinase, and the lipoxygenases, promote the formation of a tumor immunosuppressive microenvironment and facilitate immune escape (222–225). In this sense, Wu and collaborators (226) revealed that the immunosuppressive state of TAMs is modulated by long-chain unsaturated FA metabolism, specifically that of oleic acid. By targeting crucial organelles in myeloid TAM, with chemical inhibitors, TAM polarization was blocked *in vitro*, and tumor growth was inhibited *in vivo* (226). Likewise, Goossens et al. (227) characterized TAM in a metastatic ovarian cancer mouse model, revealing that ovarian cancer cells induce membrane-cholesterol efflux and deplete lipid rafts from macrophages. Enhanced cholesterol efflux facilitates IL4-mediated reprogramming, inhibiting IFNγ-induced gene expression. Deleting ABC transporters, which mediate cholesterol efflux, reverses the tumor-promoting functions of TAM, reducing tumor progression. These findings highlight a previously unrecognized role for membrane-cholesterol efflux in driving TAM-mediated tumor progression, suggesting a potential novel anti-tumor therapeutic approach (179). Finally, Prima and colleagues (228) showed that the COX-2/mPGES1/PGE_2_ pathway plays a role in regulating PD-L1 (programed death ligand-1, CD274) expression in tumor-infiltrating myeloid cells. Consequently, altering PGE_2_ metabolism in the tumor microenvironment could help reduce immune suppression in the tumor host.

## 4 Conclusions and perspectives

Despite the significant progress in macrophage biology, great challenges remain to precisely define the polarization states of macrophages and identify robust biomarkers beyond the classic ones available today. The characteristic plasticity of these cells implies additional experimental complexity, since they exhibit very diverse phenotypes under normal and pathological conditions. However, this biological characteristic represents at the same time an extraordinary possibility of therapeutic intervention in an enormous number of pathologies. The lipidomic analyses of macrophages stress that, throughout the polarized activation process, several key enzymes involved in lipid biosynthetic pathways are induced, expressed, and activated to different degrees. Therefore, the changes in the levels of the different lipid classes are the consequence of highly orchestrated enzymatic processes, many of which are still not fully characterized or understood. Clearly, further research will be needed to understand how signaling pathways and enzymatic processes coordinate the reshaping of macrophage lipid composition with different phenotypes under different stimuli, which is crucial for their function. To this end, efforts should be made to integrate multi-omics approaches, including transcriptomics, proteomics, and metabolomics, to comprehensively characterize macrophage lipid metabolism within the whole polarization landscape, and identify suitable biomarkers. In this regard, several studies have employed single-cell and spatial transcriptomic techniques, often integrated with lipidomic analyses, to investigate lipid metabolism in relation to macrophage phenotypes. For instance, a study utilizing single-cell RNA sequencing identified distinct macrophage populations with unique lipid metabolism profiles, shedding light on their roles in various physiological and pathological contexts (229). Another research effort combined single-cell sequencing with spatial transcriptomics to explore the tumor microenvironment, revealing that M1 macrophages can suppress lipid metabolism in pancreatic adenocarcinoma cells, thereby affecting tumor progression (230). Additionally, spatial transcriptomics has been applied to examine perivascular macrophages, uncovering alterations in lipid metabolism that influence their function and contribution to disease states (231). These studies collectively demonstrate the utility of advanced transcriptomic and metabolomic approaches in elucidating the complex relationship between lipid metabolism and macrophage phenotypes. These advances will improve our understanding of macrophage biology and facilitate the discovery of targeted therapies for various diseases.

## Glossary

AA: arachidonic acid
ACLY: ATP-citrate lyase
ACC: acetyl-CoA carboxylase
ACSL: acyl-CoA synthetase, long chain
BMDM: bone marrow-derived macrophages
COX: cyclooxygenase
CPT1: carnitine palmitoyltransferase 1
DAG: diacylglycerol
DHA: docosahexaenoic acid
EPA: eicosapentaenoic acid
ETC: electron transport chain
FABP4: fatty acid binding protein 4
FA: fatty acid
FAO: fatty acid β-oxidation
FAS: fatty acid synthase
GM-CSF: granulocyte-macrophage colony-stimulating factor
HMG-CoA: 3-hydroxy-3-methylglutaryl-CoA
IFNγ: interferon γ
IL: interleukin
KLA: Kdo2 lipid A
LDL: low-density lipoprotein
LOX: lipoxygenase
LPC: lysophosphatidylcholine
LPS: lipopolysaccharide
LT: leukotriene
LXR: liver X receptor
M1/M2: macrophage activation phenotypes (pro-inflammatory and anti-inflammatory)
M-CSF: macrophage colony-stimulating factor
MyD88: myeloid differentiation primary response 88
NADPH: nicotinamide adenine dinucleotide phosphate (reduced)
NF-κB: nuclear factor kappa-light-chain-enhancer of activated B cells
NO: nitric oxide
OXPHOS: oxidative phosphorylation
PA: phosphatidic acid
PAF-AH: platelet-activating factor acetyl hydrolases
PC: choline-containing glycerophospholipids
PE: ethanolamine-containing glycerophospholipids
PG: prostaglandin
PI: phosphatidylinositol
PLA_2_: phospholipase A_2_
PS: phosphatidylserine
PUFA: polyunsaturated fatty acid
ROS: reactive oxygen species
SCFA: short-chain fatty acid
SREBP1: sterol regulatory element-binding protein 1
TAG: triacylglycerol
TAM: tumor-associated macrophage
TCA: tricarboxylic acid cycle
Th: T-helper lymphocytes
TLR: Toll-like receptor
TRAF6: TNF receptor-associated factor 6
TX: thromboxane.
